# Evaluation of an EPID in vivo monitoring system using local and external independent audit measurements

**DOI:** 10.1002/acm2.13822

**Published:** 2022-11-10

**Authors:** Samuel Ramalho Avelino, Juliana Rosada Dias, Taís Marques Peron, Gabriel Souza Vidal

**Affiliations:** ^1^ VITTA Radiotherapy Center Brasília‐DF Brazil; ^2^ Department of Radiation Oncology Stephenson Cancer Center University of Oklahoma Oklahoma City United States

**Keywords:** EPID, in vivo dosimetry, quality assurance

## Abstract

**Purpose:**

The aim of this work was to evaluate the SunCHECK PerFRACTION, the software for in vivo monitoring using EPID images.

**Materials/Methods:**

First, the PerFRACTION ability to detect errors was investigated simulating two situations: (1) variation of LINAC output and (2) variation of the phantom thickness. An ionization chamber was used as reference to measure the introduced dose variations. Both tests used EPID in integrated mode (absolute dose). Second, EPID measurements in integrated mode were carried out during an independent Brazilian governmental audit that provided four phantoms and TLDs. PerFRACTION calculated the absolute dose on EPID plane, and it compared with predicted calculated dose for every delivered plan. The dose deviations reported using PerFRACTION were compared with dose deviations reported by the independent audit. Third, an end‐to‐end test using a heterogeneous phantom was performed. A VMAT plan with EPID in cine mode was delivered. PerFRACTION calculated the mean dose on CBCT using EPID information and log files. The calculated doses at four different points were compared with ionization chambers measurements.

**Results:**

About the first test, the largest difference found was 1.2%. Considering the audit results, the variations detected by TLD measurements and by PerFRACTION dose calculation on EPID plane were close: 12 points had variations less than 2%, 2 points with variation between 2% and 3%, and 2 points with deviations greater than 3% (max 3.7%). The end‐to‐end tests using a heterogeneous phantom achieved dose deviation less than 1.0% in the water‐equivalent region. In the mimicking lung region, the deviations were higher (max 7.3%), but in accordance with what is expected for complex situations.

**Conclusion:**

The tests results indicate that PerFRACTION dose calculations in different situations have good agreement with standard measurements. Action levels were suggested for absolute dose on EPID plane as well as 3D dose calculation on CBCT using PerFRACTION.

## 1 INTRODUCTION

In vivo dosimetry (IVD) is a recommended procedure in quality assurance protocols^1^, ^2^, ^3^, ^4^ and is mandatory in some countries such as Sweden and France.^5^, ^6^ EPID measurements can be used to perform IVD.^6^ This can be done by comparing measurements to forward 2D dose calculations in the EPID plane^7^, ^8^ or by a back‐projection method that reconstructs the 3D dose on planning CT or CBCT using the primary dose to the EPID.^8–1^, ^1^

Regardless of the technique, the benefits to using IVD with EPID have been described by many studies.^8^, ^12^, ^13^, ^14^ IVD with EPID can accurately detect machine‐, plan‐, and patient‐related errors.^8^

The number of radiotherapy facilities performing IVD is expected to increase. When the concept of using EPID to perform IVD was first developed, there were no commercial software applications available to manage the EPID data. This was considered the main drawback about IVD using EPID.^6^ However, this issue is no longer a barrier to IVD implementation now given at least six commercial solutions for using EPID data for IVD.^8^

The aim of our work is to evaluate the SunCHECK PerFRACTION (Sun Nuclear Corporation, Melbourne, FL, USA) software for in vivo monitoring through end‐to‐end tests, by carrying out comparison measurements with TLDs and ionization chambers in six different phantoms. The PerFRACTION mode called “Fraction N,” which was developed for IVD or in vivo monitoring, was used.

PerFRACTION uses EPID measurements to calculate the planar dose in the portal (forward 2D dose calculation), as well as LINAC treatment log files to calculate the volumetric dose in the planning CT or in the treatment session CBCT.^15^, ^16^ PerFRACTION was implemented with a superposition/convolution GPU‐accelerated dose computation algorithm.^17^ The dose calculation consists of fluence calculation, total energy released per unit mass (TERMA), and superposition.^15^ In this dose calculation validation work, the authors found an average dose difference between the calculation and ionization chamber measurements of −0.3% ± 0.8% (1SD) in water‐equivalent phantoms. Moreover, the accuracy achieved in most planar patient‐specific tests was within the level expected in clinical routine using gamma‐index analyses (≥95% passing rate: 3%/2 mm, global, threshold 10%).^15^, ^17^

**FIGURE 1:**
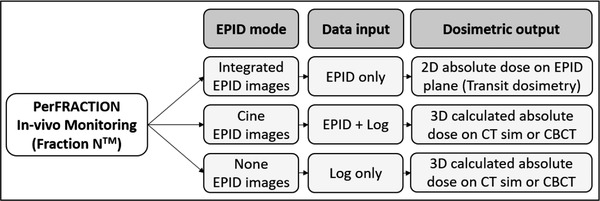
PerFRACTION modes for in vivo monitoring and dosimetry. The system considers the type of EPID images acquired. If no EPID images are available, only log files are used.

About “Fraction N” mode, if the EPID image is acquired using integrated (dosimetry) mode during the treatment, PerFRACTION converts the image to absolute planar dose for comparison to a predicted planar dose generated from the RT Plan and CT dataset. In this case, the 3D dose distribution is also calculated on the planning CT or in the CBCT using the treatment log files.^15^ If the image is acquired in cine mode, PerFRACTION uses for 3D dose calculation the EPID MLC projection of each frame in addition to other treatment logs (output, dose rate, gantry position etc.). This method is known as EPILOG.^14^, ^18^ PerFRACTION can also calculate dose in the planning CT or CBCT even without the EPID exposure, using only the treatment log files (in this case, including MLC positions from the log files). Figure 1 illustrates an overview about the calculation modes available on “Fraction N” module in PerFRACTION.

PerFRACTION is already being used with success for IVD in clinical practice.^12^, ^17^ However, it is still important to assess the capability and precision to detect errors using an IVD program based on PerFRACTION. Olaciregui‐Ruiz et al.^8^ suggested, in a review article, that vendors and users should make an effort to evaluate uncertainties, sensitivity, and specificity of IVD solutions in order to define reasonable and relevant action levels for clinical practice. Mijnheer et al. said that it is very important to define action levels that are feasible from a workload perspective, as well as being capable of detecting clinically relevant errors.^14^ Understanding uncertainties in controlled tests is crucial to define reliable action levels for an IVD program.

Some studies investigated PerFRACTION in case‐controlled tests. They evaluated sensitivity and uncertainties for machine‐,^19^, ^20^ plan‐,^16^, ^20^, ^21^ and patient‐related errors.^18^, ^19^, ^20^

This work contributes by expanding the set of case tests to which PerFRACTION was subjected. It is important to highlight that six different phantoms were used in this study, including one with a high level of heterogeneity. Tests involving dose calculations on CBCT based on log files and MLC positions detected with EPID were also performed. No studies taking into account in vivo monitoring in this way were identified. Moreover, tests were carried out during an independent audit by a Brazilian governmental agency, increasing the level of confidence of our results. Finally, our study concurs with recommendations made by Olaciregui‐Ruiz et al.^8^ that users and vendors still need to test IVD solutions extensively in order to define action levels for daily practice. The action levels in an IVD program should be in accordance with the uncertainties involved in this complex scenario.

## 2 METHODS

In order to evaluate all calculation possibilities available in the “Fraction N” mode of the PerFRACTION system, end‐to‐end tests were performed focusing on IVD using EPID measurements. The tests used 6 and 10 MV photon beams of a Varian TrueBeam, v2.7, (Varian, Palo Alto, CA, USA) linear accelerator and Varian Eclipse TPS (Varian, Palo Alto, CA, USA). The TPS was upgraded during the progress of this study. The TPS version and algorithm used in each test are shown in the following subsection. A source‐EPID distance of 150 cm was used in all cases; the same used for commissioning PerFRACTION.

### 2.1 PerFRACTION—“Fraction N” with EPID

“Fraction N” was investigated for absolute dose calculation on the EPID plane (integrated images). In this mode, the software additionally calculates 3D dose distribution on CT‐plan or CBCT using the log files of that beam delivery.

In addition, “Fraction N” was evaluated for 3D dose calculation on CBCT using log files (excluding MLC positions) + MLC positions detected with EPID. Sun Nuclear calls this method EPILOG. It is important to highlight that in this case, the EPID intensity is not considered for the dose calculation. The software uses MLC projection on each cine frame to identify MLC positions for forward‐projected 3D dose calculation in conjunction with dose outputs from the log files.

### 2.2 Preliminary tests

**FIGURE 2:**
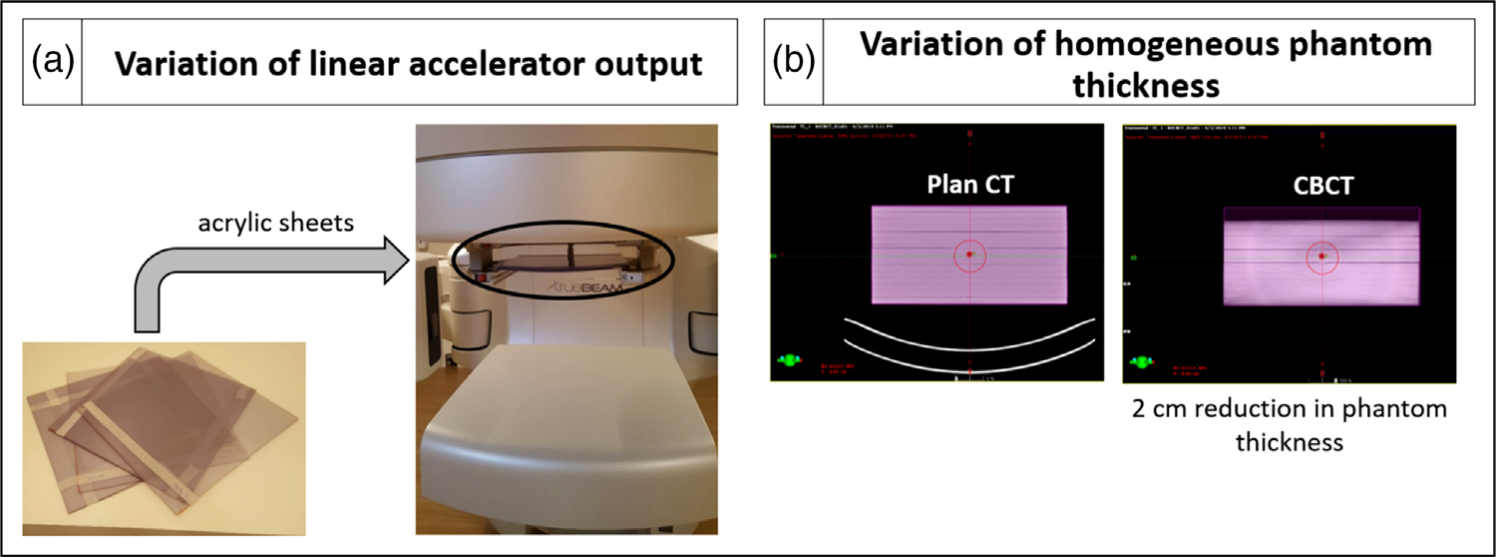
(a) Setup used to attenuate the beam of the linear accelerator with acrylic sheets; (b) planning CT of the homogeneous phantom (on the left) and CBCT of the phantom with the thickness reduced by 2 cm (on the right)

The PerFRACTION ability to detect errors was preliminarily investigated simulating two scenarios—(1) variation of linear accelerator output: acrylic sheets were attached to the collimator for beam attenuation; (2) variation of the phantom thickness: reduction of 2 cm in the homogeneous phantom thickness during the irradiation. Figure 2 illustrates the setup of each scenario.

A Farmer ionization chamber was used as reference to measure the introduced dose variations (three dose measurements were performed in each test). Both sensitivity tests used EPID in integrated mode. Although PerFRACTION can calculate the dose on EPID surface, the dose calculated on central axis of the EPID plane was compared with predicted dose (without beam and phantom modifications). Preliminary tests were performed considering square open fields (homogeneous dose in the exposed EPID area); therefore, a dose point approach is suitable. The dose difference in the EPID central axis was directly compared with the ionization chamber measurements deviation in both preliminary tests.

In the case of phantom thickness variation, the 3D dose calculation using the treatment log files in the CBCT was additionally analyzed. The mean dose calculated by PerFRACTION in the ionization chamber sensitive volume (previously contoured in the phantom CT) was compared with its measured dose.

### 2.3 End‐to‐end tests during an independent audit (integrated EPID images)

Transmission dosimetry with EPID has the advantage of not interfering with treatment/test dose distributions. Therefore, PerFRACTION could be tested during the annual independent auditing program provided by the Brazilian government. This voluntary program provides institutions with four phantoms and TLDs to be irradiated. Its measurements were compared with the TPS dose calculation.

**FIGURE 3:**
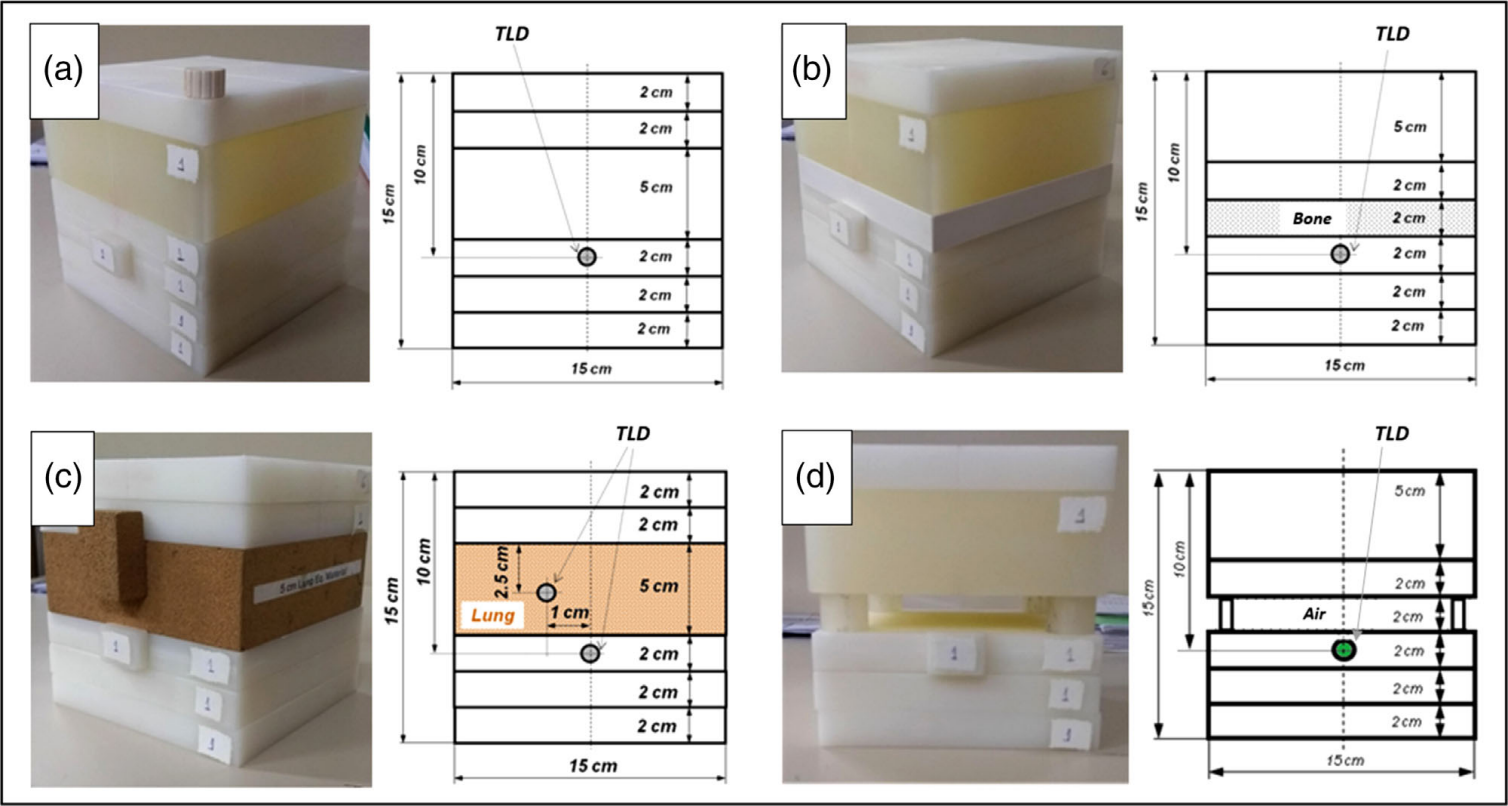
Setups used during the external audit for each phantom: (a) homogeneous phantom; (b) 01 slab mimicking bone; (c) 01 slab mimicking lung; and (d) phantom with an air region

The setup recommended by the auditing group was followed for all phantoms. It consists of an open square field delivering 200 cGy to a TLD, using monitor units calculated by the TPS. For this test, the analytical anisotropic algorithm (AAA) of Eclipse TPS v15.1 was used. The AAA is a kernel‐based convolution/superposition method. The AAA applies density scaling of Monte Carlo–derived kernel for a homogeneous medium.^22^ Considering measurements as reference, Fogliata et al. reported an accuracy of 1% for output factors of open beams calculated using AAA. The authors showed a 1%/1 mm average accuracy in the calculated depth–dose curves.^23^ In heterogeneous regions as a thoracic phantom, Esch et al. reported that AAA can achieve 5% agreement with measurements.^24^ During beam delivery, EPID used integrated/dosimetry mode to collect images. The external independent audit sent four different phantoms: (a) homogeneous phantom; (b) 01 slab mimicking bone; (c) 01 slab mimicking lung; and (d) phantom with an air region. Figure 3 shows the position of the TLD for every phantom.

During the irradiation of the TLDs, EPID measurements (integrated mode) were carried out in order to compare the results in an indirect way (Figure 3). The EPID central axis measured dose with PerFRACTION was compared with TPS dose calculation and with TLD measurements. The audit program provides only point dose detectors (TLD) that are positioned in the central axis of the beam. The audit setup limits the possibility to fully use the EPID 2D dose measurements for comparison, but it actually has the advantage to provide dose results certified by an independent governmental audit. The audit program provides one TLD for every plan delivery (single‐dose measurement in each phantom was performed).

### 2.4 End‐to‐end tests using a heterogeneous phantom (cine EPID images)

**FIGURE 4:**
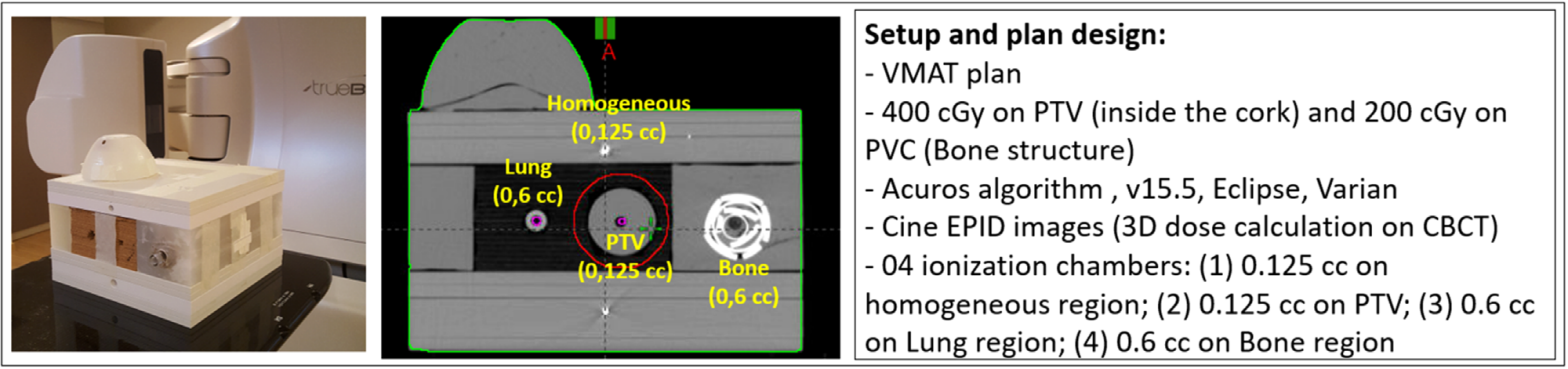
Setup used during the end‐to‐end test to compare doses measured using four ionization chambers with mean doses calculated on sensitive volumes of the detectors by PerFRACTION. EPILOG method was used to calculate the dose on CBCT image.

An end‐to‐end test was performed using an internally developed heterogeneous phantom, which simulates lung (cork), bone (PVC), and water‐equivalent medium (solid water slabs and wax). The phantom has four spots for ionization chambers (Figure 4): (1) homogeneous region (IC volume: 0.125 cm^3^), (2) PTV region (IC volume: 0.125 cm^3^), (3) lung region (IC volume: 0.6 cm^3^), and (4) bone region (IC volume: 0.6 cm^3^). A VMAT plan was used for phantom irradiation with the EPID in cine mode. The plan was designed to deliver 400 cGy to the PTV (involving wax cylindrical volume inside the cork) and 200 cGy to the structure simulating bone (PVC). For this end‐to‐end case test, the Acuros algorithm of Eclipse TPS v15.5 was used. Acuros explicitly solves the Linear Boltzmann Transport Equations by numerical methods, similar to the Monte Carlo methods. Compared with AAA, Acuros XB improves accuracy in the presence of inhomogeneity.^22^ Mean dose was calculated inside the sensitive volume of each ionization chamber by PerFRACTION and was compared with the actual dosimeter measured doses for a single‐dose delivery.

Table 1 shows the characteristics of all tests performed. It demonstrates a comprehensive evaluation of the “Fraction N” mode of PerFRACTION.

**TABLE 1:** Overview of all tests performed to evaluate “Fraction N” mode of PerFRACTION

EPID mode	Type of calculation	Type of test	Type of related error	Description	Detectors
Integrated	2D dose on EPID	Sensitivity	Machine	Homogeneous phantom + modified LINAC output	Ionization chamber
Integrated	2D dose on EPID	Sensitivity	Patient	Modified homogeneous phantom thickness	Ionization chamber
Integrated	2D dose on EPID	End‐to‐end	Plan	Homogeneous phantom	External audit with TLD + local TPS
Integrated	2D dose on EPID	End‐to‐end	Plan	Phantom with 01 slab simulating bone	External audit with TLD + local TPS
Integrated	2D dose on EPID	End‐to‐end	Plan	Phantom with 01 slab simulating lung	External audit with TLD + local TPS
Integrated	2D dose on EPID	End‐to‐end	Plan	Phantom with 01 air region	External audit with TLD + local TPS
Integrated	3D dose on CBCT using only log files	Sensitivity	Patient	Modified homogeneous phantom thickness	Ionization chamber
Cine	3D dose on CBCT using partial log files + MLC positions detected with EPID	End‐to‐end	Plan	Phantom with a high level of heterogeneity	Four ionization chambers + TPS

## 3 RESULTS

### 3.1 LINAC output variation

PerFRACTION detected variations of linear accelerator output for 6 and 10 MV during IVD output preliminary test. The dose reductions, introduced by acrylic sheets, were 1.6% and 3.0% for 6 and 10 MV, respectively, when measured with ionization chambers. Central axis dose values sampled from the PerFRACTION measured EPID dosimetry indicated dose reductions of 2.0% and 1.8% for 6 and 10 MV, respectively (Table 2).

**TABLE 2:** Test results of PerFRACTION sensitivity related to variation of linear accelerator output and variation of the phantom thickness for 6 and 10 MV

Energy	Sensitivity test	Calculation mode	Expected dose deviation (%)	PerFRACTION dose deviation (%)
6 MV	Modified LINAC output	2D dose on EPID plane	1.6	2.0
	Modified phantom thickness	2D dose on EPID plane	5.5	5.8
	Modified phantom thickness	3D CBCT dose calculation using log files	5.5	4.9
10 MV	Modified LINAC output	2D dose on EPID plane	3.0	1.8
	Modified phantom thickness	2D dose on EPID plane	4.3	4.8
	Modified phantom thickness	3D CBCT dose calculation using log files	4.3	3.5

*Note*: The expected dose deviations were obtained using ionization chamber to detect the intentional beam and phantom modifications applied.

### 3.2 Phantom thickness reduction

Analogous results were obtained simulating the variation of patient anatomy. After removing a 2 cm phantom slab, the ionization chambers measured an increased dose of 5.5% and 4.3% for 6 and 10 MV, respectively. For the same delivered beams, PerFRACTION calculated an increased dose on the central axis of the EPID plane of 5.8% and 3.5% for 6 and 10 MV, respectively (Table 2).

Regarding phantom thickness variation, PerFRACTION also calculated 3D dose in the CBCT using the treatment log files acquired during the dose delivered in the previous test. PerFRACTION calculated an increased dose on the ionization chamber sensitive volume of 4.9% and 4.8% for 6 and 10 MV, respectively (Table 2).

### 3.3 Measured dose during an independent audit (integrated EPID images)

The independent external audit reported the dose differences from TLDs and the predicted doses by the TPS for every phantom. For 6 MV photons beam, in the homogeneous phantom, the TLD dose difference from TPS was −1.2%. PerFRACTION 2D dose calculation on EPID plane completely agree with predicted dose by TPS. No deviation was observed for this phantom. In the phantom with 1 slab simulating bone, the TLD dose difference from TPS was 2.9%. PerFRACTION 2D dose calculation on EPID plane was 3.7% different from TPS. In the phantom with 1 slab simulating lung, the TLD dose difference from TPS was −2.1%. In this case, PerFRACTION 2D dose calculation on EPID plane was −1.7% different from TPS. In the phantom with 1 air region, the TLD dose difference from TPS was −1.3%, whereas PerFRACTION 2D dose calculation on EPID plane was −1.7% different from TPS.

For 10 MV photon beam, in the homogeneous phantom, the TLD dose difference from TPS was −1.5%. In this case, PerFRACTION 2D dose calculation on EPID plane was 0.3% different from TPS. In the phantom with 1 slab simulating bone, the TLD dose difference from TPS was −0.1%. PerFRACTION 2D dose calculation on EPID plane was 3.2% different from TPS for this phantom. In the phantom with 1 slab simulating lung and with 1 air region, the TLD dose difference from TPS was −0.4% and −1.6%, respectively. PerFRACTION 2D dose calculation on EPID plane completely agrees with TPS‐predicted dose for these both phantoms.

Using TPS calculations as reference, the mean dose deviation between TLD and EPID measurements was 1.2% ± 1.0%. Table 3 shows the deviations of measurements by the TLDs, as well as those of dose calculated by PerFRACTION on the EPID, with respect to the TPS‐predicted doses.

**TABLE 3:** TLD dose deviations from TPS measured during an external audit using four different phantoms for 6 and 10 MV photon beams

Energy	Phantom	TLD–TPS dose deviation (%)	PerFRACTION–TPS dose deviation (%)	TLD–PerFRACTION dose deviation (%)
6 MV	Homogeneous	−1.2	0.0	1.2
	Bone	2.9	3.7	0.8
	Lung	−2.1	−1.7	0.4
	Air region	−1.3	−1.7	0.4
10 MV	Homogeneous	−1.5	0.3	1.8
	Bone	−0.1	3.2	3.3
	Lung	−0.4	0.0	0.4
	Air region	−1.6	0.0	1.6
Mean deviation (%):				1.2 ± 1.0

*Note*: PerFRACTION dose deviation from TPS using integrated EPID measurements during every external audit beam exposure.

### 3.4 3D dose calculated in a heterogeneous phantom CBCT (cine EPID images)

The end‐to‐end test using an internally developed heterogeneous phantom provided results in four different regions. Using ionization chamber measurements as reference, Table 4 shows the dose deviations calculated by PerFRACTION using the EPILOG method in the CBCT image. The dose differences in PTV, lung, bone, and homogeneous regions were 5.9%, 5.8%, 6.9%, and −0.1% for 6 MV photons beam, respectively. The dose differences in PTV, lung, bone, and homogeneous regions were 4.7%, 3.3%, 7.3%, and −0.9% for 10 MV photons beam, respectively. Considering all phantom regions, the mean dose deviation between ionization chamber measurements and PerFRACTION dose calculations was 4.4% ± 2.7%.

**TABLE 4:** Dose measurements using ionization chambers in four regions at a heterogeneous phantom were used as reference

Energy	Measurement region	Reference dose (cGy)	PerFRACTION dose (cGy)	Deviation (%)
6 MV	PTV	400.3	424	5.9
	Bone	208.0	220	5.8
	Lung	180.6	193	6.9
	Homogeneous	215.3	215	−0.1
10 MV	PTV	399.3	418	4.7
	Bone	197.4	204	3.3
	Lung	166.9	179	7.3
	Homogeneous	203.9	202	−0.9
Mean deviation (%):				4.4 ± 2.7

*Note*: PerFRACTION calculated the mean delivered dose in the sensitive volume of all detectors using cine EPID measurements and machine log files (EPILOG method). The dose deviation is presented.

## 4 DISCUSSION

There is a lack of studies and protocols to define tolerance levels for 2D/3D dose calculation in IVD. It is even rarer to find studies suggesting metrics to evaluate IVD based on EPID measurements. In order to analyze the results, uncertainties reported in studies that evaluated dose measurements in similar conditions were taken into account.

### 4.1 Preliminary tests (LINAC output and phantom thickness)

Zhuang and Olch performed many tests to evaluate PerFRACTION sensitivity related to machine and patient setup errors.^19^ PerFRACTION detected machine output error within 0.2% and setup errors as small as 1 mm and 0.5°. Doolan et al. evaluated PerFRACTION sensitivity extensively using integrated EPID images.^20^ Variations in the beam output could be detected within 0.3%. Regarding patient variation, they reported that 5 mm reduction in VMAT plans and 10 mm in conformal plans could be detected.

In both studies mentioned earlier, the tests were performed with image (baseline) to image comparisons and not with the predicted image calculated using the transit absolute dosimetry calibration. The calibration process introduces its own inherent uncertainty. Sensitivity might differ when comparing to a baseline image versus a predicted image. In our study, the trade‐off for the slightly larger uncertainty from EPID calibration is that predicted images have the advantage of being able to validate the first fraction against the setup from the planning CT. Bojechko et al.^13^ found a significant portion of errors detected during that first fraction.

The preliminary tests, in this current study, demonstrated that PerFRACTION accurately detected variations of linear accelerator output in the in vivo monitoring. Analogous results were obtained for the simulated patient thickness variation. There is an excellent agreement between ionization chamber measurements and EPID measurement at central axis. The largest difference identified was 1.2%. These simplified preliminary tests could demonstrate that PerFRACTION is able to detect patient and machine errors in a satisfactory way.

### 4.2 System performance during an independent audit (integrated EPID images)

Studies that investigated PerFRACTION using integrated EPID images for machine‐, plan‐, and setup‐related errors as an end‐to‐end test were not found.

Hsieh et al. evaluated setup‐related errors using 2D dose calculation on the EPID plane.^25^ They used gamma pass rate and dose percent difference to investigate shifts and rotations. The authors suggest that gamma metrics have insufficient sensitivity to detect inter‐ and intra‐fraction setup errors meeting or exceeding commonly used PTV margins. The DTA component of gamma masked the introduced setup errors. They recommend metric percent difference as an alternative, which together with stringent pass/fail criteria, has high sensitivity to the stated clinical setup error detection goal.

In a similar way, considering integrated EPID images, Doolan et al. reported that VMAT beams were less sensitive to patient misalignments, with a shift of 10 mm only detectable once a strict criterion of 1% dose difference was applied.^20^

A comprehensive and innovative method to test an IVD software based on integrated EPID images was used in our study. Independent audit is a powerful method to detect deviations on a QA program. The independent audit process increases the level of confidence of these tests. Ibbott et al. report that surprising results occurred during an external audit using anthropomorphic head‐and‐neck phantom. A significant number of radiotherapy facilities could not fulfill the clinically acceptable criteria using the gamma index method of evaluation (dose difference of ±7% or distance‐to‐agreement of 4 mm).^26^ This study contributes to highlighting the importance of independent audits and IVD.^6^

The independent audit using TLD measurements and four different phantoms to verify TPS dose calculation is a traditional QA barrier used extensively for radiotherapy departments in Brazil. Using the setup of this independent audit, we could find that variations detected by TLD measurements and by PerFRACTION dose calculation on EPID plane were very close. The mean dose deviation between TLD and PerFRACTION was 1.2% ± 1.0% (Table 3). Among the 16 points analyzed (TLDs and EPID): 12 points had variations less than 2%, 2 points with variation between 2% and 3%, and only 2 points with deviations greater than 3%. The major deviations were observed for PerFRACTION calculation in the phantom region that simulated bone. However, for 6 MV, the TLD also showed a high deviation to TPS, similar to those detected by PerFRACTION. This finding suggests that an investigation of TPS dose calculation in high‐density medium should be performed.

### 4.3 System dose calculation in a heterogeneous phantom CBCT (cine EPID images)

Sait et al. conducted a set of tests to evaluate PerFRACTION 3D dose calculation based on log files and cine EPID images.^21^ They assessed errors related to plan and patient setup. Phantoms received repetitive fractions of volumetric modulated arc therapy, simulating prostate treatment. There was concordance between ionization chamber measurements and PerFRACTION 3D absolute point dose calculation (mean dose deviation of 0.1%). Close agreement was also obtained between *X*‐ and *Y*‐axis dose profiles with PerFRACTION‐calculated doses, MapCHECK‐measured doses, and planning system–predicted doses (max dose deviation of 3.9% between MapCHECK‐measured doses and PerFRACTION 3D dose calculation). Both case scenarios used homogeneous phantoms.

Kadoya and colleagues performed tests to specifically evaluate PerFRACTION 3D dose calculation.^16^ They focused on plan‐related errors of five prostate cancer patients who were previously treated. Log files and cine EPID images were acquired for each fraction. The treatment planning dose was recalculated on homogeneous and heterogeneous phantoms. Dose difference at isocenter between measurement and reconstructed dose for two software programs was within 3.0% in both homogeneous and heterogeneous phantoms. In a particular test that considered the use of skip arc plans, the dose deviations between measurements and reconstructed were higher (up to 4.5%).

The tests performed in this study could be considered extremely complex, because the 3D dose was calculated on a very heterogeneous phantom CBCT (mimicking lung and bone regions). The mean dose deviation between dose measurements and PerFRACTION dose calculation was 4.4% ± 2.7% (Table 4). Considering the results in the homogenous region of the phantom, a good agreement for both energies was observed (deviation less than 1.0%). In other regions, the deviations were higher; however, deviations up to 8.0% occurred in previous studies^6^, ^27^ that investigated similar complex situations (not using PerFRACTION). Mans et al. suggested that the use of treatment log file in addition to dose calculation on CBCT is close to an end‐to‐end check of the planned and delivered dose distribution.^28^

### 4.4 Action levels in clinical practice

It is important to highlight that desired and achievable uncertainties could diverge for heterogeneous regions.^27^ Thwaites reported that an additional uncertainty of 5% should be considered in the global treatment uncertainty achievable for lung region. When considering IVD uncertainties, all uncertainties in the radiotherapy process impact the global evaluation errors. Thwaites estimated an overall cumulative uncertainty on delivered patient dose about 8% for lung treatments. In our study, three different phantoms with low density regions were used. The results are within the achievable uncertainty suggested by Thwaites for heterogeneous situations.

In regard to IVD uncertainties, Mijnheer et al. mentioned that often an action level of 5% is used for simple treatments and 7% for situations where measurement complications exist (as breast treatments or in wedged beams).^6^

Esposito and colleagues studied the dose delivery accuracy of stereotactic body radiation therapy (SBRT). In phantom, accuracy of point dosimeters used for VMAT and SBRT treatments ranged from 2% for plastic scintillators to 8% for TLD.^29^ In this review, the authors state that EPID proved to have high sensitivity in detecting anatomical variations and LINAC parameter errors, such as leaf position and collimator gantry position errors.

This study has the limitation that intentional shifts and rotations on phantom setup were not investigated. However, patient misalignment might be detected with more efficiency in a daily IGRT routine, instead of using an IVD method.^30^ Another limitation is the use of single measurement for each test. However, considering that the results presented are within an acceptable clinical tolerance uncertainty, they can be used as reference for an initial QA program based on PerFRACTION.

In summary, the authors recommend the use of dose difference metric instead of gamma function metric for homogeneous dose distributions. The results suggest that an action level of 5% could be used for situations involving IVD based on integrated EPID images (absolute dose on EPID plane). For PerFRACTION mode using cine EPID images (3D dose calculation), action levels of 3% and 8% could be used for homogeneous and heterogeneous regions, respectively. The authors suggest that PerFRACTION users register their own patients’ results to move forward action levels based on a statistical process control approach.

## 5 CONCLUSION

Challenging tests were performed using six different phantoms (in particular, one very heterogeneous phantom), dose calculations on CBCT, and measurements with TLD and ionization chambers. This study was the first to evaluate PerFRACTION during a certified independent audit.

The results indicate that PerFRACTION dose calculations in different situations have good agreement with standard measurements. Therefore, considering the variety and complexity of the tests in this study, the PerFRACTION system can be considered effective to increase patient safety through IVD and in vivo monitoring. The results of this study could be used in an initial QA program based on PerFRACTION. A dose difference metric with an action level of 5% could be used for IVD based on integrated EPID images. For PerFRACTION mode using cine EPID images, distinct action levels could be used for homogeneous and heterogeneous regions (3% and 8%, respectively). The authors suggest that a statistical process control approach could be further used to define action levels based on clinical patient measurements.

The next steps for this study will focus on the analysis of 2D dose distributions of modulated plans using integrated EPID images, and evaluation of action levels for DVH metrics in PerFRACTION 3D dose calculation mode.

## CONFLICT OF INTEREST

VITTA Radiotherapy Center is a reference site for Sun Nuclear Corporation (SNC). However, this study was not supported by SNC. The methods/results presented did not have any conflict.

## AUTHOR CONTRIBUTION

Samuel Ramalho Avelino: substantial contributions to the conception and design of the work; acquisition, analysis, and interpretation of data; drafting the work; and final approval of the version to be published

Juliana Rosada Dias: substantial contributions to the conception and design of the work; acquisition, analysis, and interpretation of data; drafting the work; and final approval of the version to be published

Taís Marques Peron: substantial contributions to the conception and design of the work; acquisition, analysis, and interpretation of data; drafting the work; and final approval of the version to be published

Gabriel Souza Vidal: substantial contributions to interpretation data for the work; revising it critically; and final approval of the version to be published
